# Mobile App-based documentation of patient-reported outcomes — 3-months results from a proof-of-concept study on modern rheumatology patient management

**DOI:** 10.1186/s13075-021-02500-3

**Published:** 2021-04-19

**Authors:** Jutta G. Richter, Christina Nannen, Gamal Chehab, Hasan Acar, Arnd Becker, Reinhart Willers, Dörte Huscher, Matthias Schneider

**Affiliations:** 1 Policlinic for Rheumatology & Hiller Research Unit for Rheumatology, Medical Faculty, Heinrich-Heine-University Duesseldorf, University Clinic, Moorenstr, 5, 40225 Duesseldorf, Germany; 2 Ortenau Klinikum Offenburg-Kehl, Offenburg, Germany; 3 Institute of Biometry and Clinical Epidemiology, and Berlin Institute of Health, Charité – Universitaetsmedizin, Berlin, Germany; 4 German Rheumatism Research Center Berlin, Berlin, Germany

**Keywords:** Rheumatoid arthritis, Electronic patient reported outcomes, Mobile Apps, Digital health

## Abstract

**Background:**

Mobile medical applications (Apps) offer innovative solutions for patients’ self-monitoring and new patient management opportunities. Prior to routine clinical application feasibility and acceptance of disease surveillance using an App that includes electronic (e) patient-reported outcome measures (PROMs) warrant evaluation. Therefore, we performed a proof-of-concept study in which rheumatoid arthritis (RA) patients used an App (RheumaLive) to document their disease.

**Methods:**

Accurate PROM reporting via an App in comparison to paper-based versions was investigated to exclude media bias. Sixty participants recruited from 268 consecutive RA outpatients completed paper-based and electronic PROMs (Hannover Functional Questionnaire/derived HAQ; modified RA disease activity index) using the App at baseline and follow-up visits. Between visits, patients used their App on their own smartphone according to their preferences. The equivalence of PROM data and user experiences from patients and physicians were evaluated.

**Results:**

Patients’ (78.3% female) mean (SD) age was 50.1 (13.1) years, disease duration 10.5 (9.1) years, and paper-based HAQ 0.78 (0.59). Mean confidence in Apps scored 3.5 (1.1, Likert scale 1 to 6). ePROMs’ scores obtained by patients’ data entry in the App were equivalent to paper-based ones and preferred by the patients. After 3 months, the App retention rate was 71.7%. Patients' overall satisfaction with the App was 2.2 (0.9, Likert scale 1 to 6). Patients and physicians valued the App, i.e., for patient-physician interaction: 87% reported that it was easier for them to document the course of the disease using the App than “only” answering questions about their current health during routine outpatient visits. Further App use was recommended in 77.3% of the patients, and according to physicians, in seven patients, the App use contributed to an increased adherence to therapy.

**Conclusion:**

Our study provides an essential basis for the broader implementation of medical Apps in routine care. We demonstrated the feasibility and acceptance of disease surveillance using a smartphone App in RA. App use was convincing as a reliable option to perform continuous, remote monitoring of disease activity and treatment efficacy.

**Trial registration:**

ClinicalTrials.gov, NCT02565225. Registered on September 16, 2015 (retrospectively registered).

## Introduction

In rheumatology, patient-reported outcome measures (PROMs) have been recognized as key outcome measures and indispensable prerequisites for improving the quality of care [1, 2]. Digital equivalents (ePROMs) have been designed to support assessments of clinical and related problems as well as the effects of treatment [3–6]. Digital health applications are recognized as important tools for modern health care systems, and encouraging prospects for the use of mobile medical applications (Apps) have been described and reviewed [7–10]. Due to the “Digital Healthcare Act” since 2020, Germany is the first country worldwide that has enabled physicians to prescribe Apps that are reimbursed by health insurances [11]. However, Apps for rheumatology are missing in that register to date.

Assessment studies in ePROMs found that computer and paper-based measures usually produce equivalent scores [12, 13]. However, a Cochrane review was not able to give clear recommendations whether Apps may influence questionnaires’ responses, and further evaluations are still recommended with a restriction if there are only minimal changes [14, 15]. Meanwhile, “Digital Healthcare Act”-associated requirements request to demonstrate equivalence in order to proof benefit [11]. Therefore, before introducing mobile Apps into clinical routine care and modern patient management concepts, PROMs obtained by paper–pencil and App versions still need to be longitudinally evaluated as data equivalence cannot be presumed [16]. In addition, patients’ capability to use a smartphone App for remote monitoring over time needs to be tested, especially in rheumatic diseases that are typically associated with functional or pain-related impairments [17].

Our aim was to prove usability and feasibility for disease surveillance and App implementation in routine care. In our proof-of-concept study, we intended to demonstrate equivalence of PROMs’ scores assessed by the RheumaLive App in comparison to paper-pencil versions in cross-sectional and follow-up assessments. RA patients’ capability to handle data entry in the smartphone App, retention rates, user experiences, and preferences of patients and physicians were investigated.

## Material and methods

The RheumaLive App was developed in German as an electronic diary for RA patients. In December 2020, it was relaunched as RheCORD. Besides diary functions, for e.g. medication, pain and morning stiffness RheumaLive implied two self-administered PROMs: the Hannover Functional Questionnaire (FFbH), which can be converted into Health Assessment Questionnaire (HAQ) values [18] that will be reported in the following, and the modified RA disease activity index questionnaire (RADAI) [19]. The RADAI—in the paper-based and electronic version—was applied to our outpatients for the first time.

Common control elements enabled data entry. Integrated algorithms calculated questionnaires’ scores. Patients’ individual follow-up data was visible to the App-user at a glance via generated PDF files customizable to personal interests.

Within our proof-of-concept study, “Mobile medically supervised patient management in rheumatoid arthritis patients using DocuMed.rh and RheumaLive App (MiDEAR),” patients and caring rheumatologists evaluated the use, usability, and feasibility of RheumaLive in routine care. MiDEAR relied on the assessment of “real-life” data and the “bring your own device” (BYOD) concept, which is an important aspect for the generalizability of the results. Patients were consecutively recruited from our single-center outpatient clinics caring for approximately 500 RA patients per year. Eligibility criteria were age above 18 years of age, diagnosis of RA (ICD-10-Code M05.* or M06.*), good German language skills, and owner of a smartphone with an operating system that was compatible for App use.

After user training, patients downloaded the App from a project-related website. They were advised to document data voluntarily on not-pre-specified intervals in their own App. They were followed-up approximately every 3 months (depending on their individually scheduled routine outpatient visits at our clinic) for 9 months. At baseline, experience with mobile devices and Apps as well as history of computer/internet use were self-documented via a modified paper-based assessment [13]. We present data from baseline and after 3 months.

Patients’ clinical and sociodemographic data were assessed according to the standard operation procedures at our clinic (e.g., Disease Activity Score of 28 joints (DAS28), medication) via our web-based patient documentation system. For further comparison, standard questions used in the national database (NDB) of the German Rheumatism Research Centre were included. Patients’ vocational education as a proxy for socioeconomic status was assessed in compliance with the NDB and the German educational system.

For the study of the media bias in PROM scores depending on the application used, RheumaLive was additionally installed on a dedicated smartphone. Patients answered both paper-based and electronic PROMs included in RheumaLive at each outpatient visit (baseline and follow-up) in random order and under similar conditions. The study coordinator gave standardized instructions at baseline, logged into the App on the clinics’ smartphone, and selected the forms. Patients answered their questions and saved the data themselves. As most patients had used the RheumaLive App between baseline and follow-up on their own device, patients were familiar with it at follow-up visits, and staff assistance was only provided on demand.

Patients and physicians reported on their IT-literacy at baseline. In follow-ups, they evaluated the App (use) assessing acceptability, usability, user satisfaction, and clinical relevance of the platform through paper-based questionnaires including i.e. questions to satisfaction, App design, and usefulness for patient-physician interaction. Where applicable Likert scales from 1 to 6 where used that based on the German school grading system.

To confirm representativeness of the study cohort for a general RA population in rheumatologic specialized care, characteristics of all RA patients of the NDB were compared to our study participants with regard to gender, age, education, comorbidities, and disease-specific scores and treatment.

We obtained each patient’s signed informed consents and ethical approval from the local ethic committee (local MiDEAR study number 4378). MiDEAR was registered to ClinicalTrials.gov (NCT02565225).

### Statistical analyzes

As MiDEAR was performed as a proof-of-concept study, the sample size was determined to consider limitations due to available funding, yet allow for statistical analysis. However, the achieved power > 0.99 for the main outcomes (data equivalence of paper-based and ePROMs) omitting adjustment for multiple testing (*α* = 0.05) showed that the number of persons included was sufficient.

Values are expressed as percentages for discrete variables, or as mean (standard deviation, SD), range, or median for continuous variables. To detect differences of paper-based PROMs and ePROMs, we tested for equivalence (“two one-sided tests” (TOST) procedure). Reported minimal clinically important differences (MCIDs) of the PROMs were used for upper and lower equivalence bounds (HAQ: 0.22 for RA in clinical trials and RADAI: 1.49) [20, 21]. Kruskal Wallis tests were performed for further group comparisons. All statistical tests were performed two-tailed, *p* values less than 0.05 were considered significant. Statistical computations used IBM SPSS Statistics version 25 for descriptive data analysis and SAS version 9.4 for test statistics.

## Results

According to the “BYOD” concept *n* = 268 consecutive RA outpatients were explored for possession of smartphones and/or tablets, *n* = 156 (58.2%) remained eligible for the study. Of those, 96 (61.5%) patients denied to participate, and the most common reasons were incompatible operating systems (29.2%), lack of interest in Apps (15.6%), and missing language skills (17.7%). Sociodemographic and clinical data of all non-participants are provided in Table 1.

**Table 1:** Sociodemographic and clinical data of participants owning a smartphone and non-participants

	Non-participants (*n* = 208)	MiDEAR participants BL (*n* = 60)	MiDEAR participant follow-up (*N* = 51)	NDB (*n* = 8481)
Age (mean (SD)) years	61.2 (14.8)	50.1 (13.1)*	49.1 (12.3)*	62.6 (14.0)
Female (*n *(%))	150 (72.1)	47 (78.3)*	40 (78.4)*	6369 (75.1)
Disease duration (mean (SD)) years	10.0 (10.1)	10.5 (9.1)*	10.5 (9.7)*	13.3 (10.3)
DAS28 (mean (SD)) baseline	2.7 (1.1)	2.6 (1.0)*	2.6 (1.0)*	2.8 (1.0)
DAS28 (mean (SD)) follow-up	n.a.	n.app.	2.5 (0.8)	n.a.
HAQ (mean (SD)) (Paper-based for MIDEAR participants)	1.21 (0.71)	0.78 (0.59)*	0.69 (0.50)*	0.88 (0.69)
HAQ (mean (SD)) follow-up (Paper-based for MIDEAR participants)	n.a.	n.app.	0.69 (0.50)	n.a.
Number of co-morbidities (median (IQR))	2 (1.0–3.0)	2 (1.0–3.8)*	2 (0–3.0) *	2 (1.0–4.0)
Education: University entrance diploma (*n* (%))	59 (28.4)	30 (50.0)*	28 (54.9)*	1120 (24.4)
Medication				
NSAIDs (*n* (%))	85 (40.9)	28 (46.7)	24 (47.1)	2782 (48.6)
Glucocorticoids (*n* (%))	121 (58.2)	30 (50.0)	26 (51.0)	2717 (47.5)
csDMARDs alone (*n* (%))	115 (55.3)	36 (60.0)	30 (58.8)	3485 (61.1)
Biological DMARD either alone or in combination with csDMARD (*n* (%))	67 (32.2)	20 (33.3)	17 (33.3)	1576 (27.6)

*n.a.* not available, *n.app.* not applicable, *values refer to baseline assessments, *BL* baseline, *NDB* national database

Sixty patients agreed to participate and to complete the paper-based and electronic PROMs in RheumaLive on the clinic’s smartphone at baseline. At the second visit after 3.5 months (median) the project had been terminated by 17 patients (28.3%); reasons were not App-dependent (e.g., time constraints, new relevant comorbidity demanding their attention). However, *n* = 51 still agreed to fill in paper-based PROMs and ePROMs.

### Clinical and sociodemographic data

Studied patients’ sociodemographic as well as clinical data are depicted in Table 1. Considering MCID, disease activity according to DAS28 remained stable between baseline and first follow-up in 74.5% (*n* = 38) of the patients.

In comparison with reference data from the NDB our participants were about 12 years younger, while non-participants were similar. Forty-seven participants were female, expected proportion from NDB was forty-five. Patients participating in MiDEAR had a by 3 years shorter disease duration, DAS28 was comparable. For HAQ, a difference was seen, while non-participants had a worse mean HAQ than reported in the NDB, participants had a slightly better HAQ. With regard to educational status, patients from our center generally seem to differ from the NDB (24%), since both for non-participants (28%) and participants (50%), the proportion with university entrance diploma was higher.

### Mobile device-related data

Operating systems on patients’ own devices were iOS (*n* = 34) and Android (*n* = 26) of different release numbers. Patients were familiar with their devices for 3.0 (2.4) years. Previous experience with mobile devices was self-rated by the patients in pre-given categories as beginners (15%, *n* = 9), laypersons (11.7%, *n* = 7), users (68.3%, *n* = 41), and professionals (5.0%, n = 3). Self-rated confidence in Apps was on average 3.5 (SD = 1.1) on a Likert scale from 1 (very high) to 6 (very low).

### Comparison of data acquisition modes

**Fig. 1:**
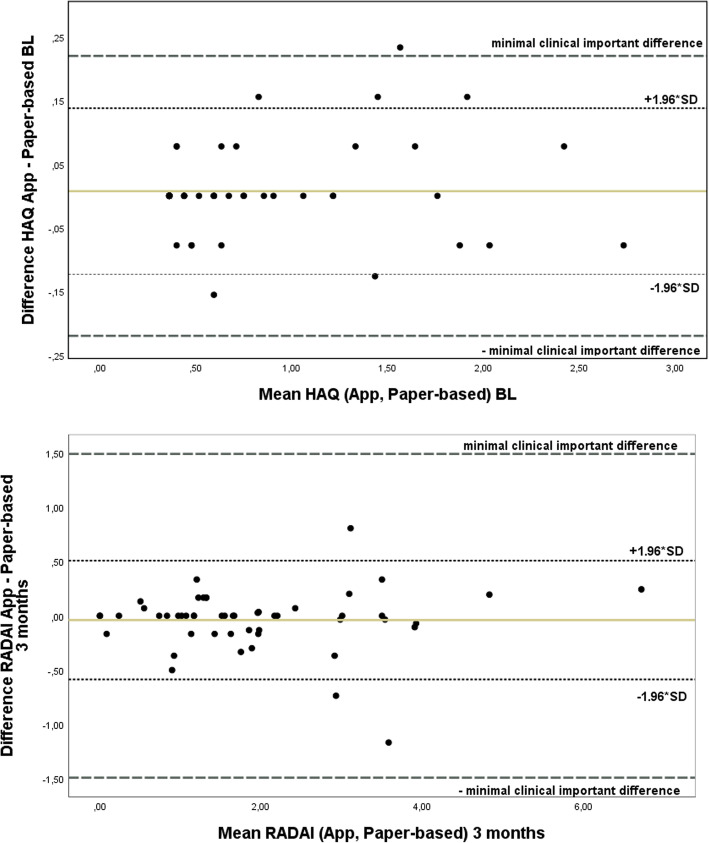
Bland-Altman plots for HAQ at baseline (BL) and RADAI after 3 months

In the entire group scores obtained by direct data entry in the App did not differ significantly from the scores obtained by the paper-based questionnaires, neither at baseline nor at the follow-up visits, see Table 2 and Fig. 1.

**Table 2:** Mean differences of derived HAQ and RADAI scores obtained by paper-respectively App-based questionnaires

PROMs score	Mean differences (SD) App – paper-based	***p*** value of equivalence tests
Baseline		
Derived HAQ (*n* = 57)	0.01 (0.07)	< 0.0001
RADAI (*n* = 58)	0.00 (0.46)	< 0.0001
Follow-up		
Derived HAQ (*n* = 51)	0.03 (0.14)	< 0.0001
RADAI (*n* = 51)	−0.04 (0.28)	< 0.0001

Analyses of score differences were performed for subgroups to investigate potential dependencies of the equality of paper-based and ePROMs. Score differences were robust for gender, age groups, and patients’ vocational education as a proxy for socioeconomic status (detailed data not shown). These findings were stable at follow-up. Furthermore, patients were divided by disease activity (DAS28 < 2.6 (baseline (BL) *n* = 33, follow-up *n* = 24), DAS28 2.6–3.1 (BL *n* = 10, follow-up *n* = 13), and DAS28 ≥ 3.2 (BL *n* = 16, follow-up *n* = 11)). Neither at baseline nor at follow-up score, differences for RADAI were significantly different between DAS28 groups (*p* > 0.05). Paper-based and ePROM score differences for HAQ were significantly different between the DAS28 groups at baseline due to higher values in patients with severe activity, but not at follow-up (*p* = 0.01 resp. *p* = 0.07). Differences between the two modes at baseline in the group with DAS28 ≥ 3.2 were still within the MCID. When patients were split up by functional capacity groups, paper-based and electronic score differences were not statistically different between the HAQ groups.

### Experiences using the App PROMs and patients’ preferences

Overall, missing data for paper-based and ePROMS were recorded in *n* = 3 patients each. Although the paper-based and ePROMs allowed missing answers, only few missing data were recorded for both the paper-based and ePROMs (maximum 4 items in *n* = 4); missing answers diminished in the follow-up visits. According to underlying algorithms, missings rarely (*n* = 1 with 4 missings in paper-based FFbH) limited the calculation of scores.

Five patients reported a lagging iPhone’s touchscreen while entering data, and no other major difficulties occurred. Only one patient felt that the RA had compromised the handling of the App between first and second visit.

At follow-up visits, 42 participants had evaluated the App. The majority (*n* = 27, 84.4%) preferred completing ePROMs rather than paper-based forms. Overall satisfaction with the App was rated as 2.2 (0.9) (mean (SD), *n* = 37, Likert scale 1–6), and the App was described by 92.3% (*n* = 36/39) as easy to use. The App design was valued as logically structured by 97.3% (*n* = 36/37), easy to understand 94.6% (*n* = 35/37), and as appealing by 83.8% (*n* = 31/37) of the patients.

Patients considered the App as useful (2.1 (1.1), mean (SD), Likert scale 1–6) i.e., for their patient-physician interaction: 63.2% (*n* = 24/38) reported that it was easier or at least partially easier (23.7%, *n* = 9/38) for them to document the course of the disease using the app than “only” answering questions about their current health during routine outpatient visits. In mean, patients filled 14.3 times (IQR 1.25–15.0) eRADAIs and 10.6 (IQR 1.5–11.5) times eFFbH respectively between baseline and follow-up. No statistically significant correlations to sociodemographic data or DAS28 were notable.

In addition, physicians evaluated the App in 22 patients. They valued the use of the App after 3 months as generally useful for the patients to document their disease progression apart from outpatient visits (2.0 (0.9) (mean (SD)), Likert scale 1–6). In 40.9% (*n* = 9), the App helped them to assess the course of the disease. The documentation with the App had a (partial) influence (*n* = 16) on the interaction during the outpatient visits. The influence was rated positively in 66.7% (*n* = 10/15). Further App use was recommended in 77.3% (*n* = 17). According to the physicians, the App contributed (partially) to an increase in the adherence to therapy in seven patients.

## Discussion

This study evaluated cross-sectional and follow-up assessments of ePROMs in an App compared to paper-based versions in patients with RA. Validation of “new” electronic versions of outcome instruments has been recommended and are required in Germany for reimbursement issues, as properties of the original instrument cannot be taken for granted and might change [2, 11, 16, 22]. Evaluation of accurate PROM reporting via an App is substantial for its use in routine care although other less arduous approaches like expert screen reviews have been proposed [15, 23, 24].

We showed that data acquisition of well-established PROMs (FFbH/HAQ; RADAI) using the RheumaLive App is a legitimate alternative to paper-pencil formats in RA patients that are willing to use a disease-related mobile App in a BYOD concept. MiDEAR produced relevant data for regulatory aspects, as e.g., medical device regulation and reimbursement issues [11, 25]. The two application modes produced concordant scores in a cross-sectional investigation but also in follow-ups even for a not yet applied PROM (modified RADAI). Thus, RheumaLive permitted regular, valid assessments of disease activity, and functional capacity even over time and is compliant with some of the later developed EULAR points to consider for mobile health applications [11, 26]. Our data are in line with a systematic review that revealed that highest pooled agreement of electronic and paper-based measures can be reported for paper-based versus touch screen assessments [14].

The App enabled RA patients to incorporate PROMs into their daily routine. As reported from other studies on ePROM assessments, our participants preferred electronic over paper-based versions [13, 22, 27]. Major App handling problems were not observed in our study, although using a relatively small touchscreen display (< 4.5 in.). Only one patient reported problems, although these could be expected more often in RA due to impaired hand function or stiffness. Larger screen sizes or the use of styluses for input can offer enhanced usability even to more functionally limited RA patients and still offer enhanced mobility. Our data is in line with results from the COmPASS study [28], and an evaluation of a touchscreen App in psoriatic arthritis patients showing that the Apps were regarded as suitable alternatives for PROM assessments [29]. De Souza et al. co-designed with patients a “hospital-specific” App that also includes the HAQ for RA patients. It was valued by them and expected to reduce non-attendance [30]. Walker et al. rated web-based patient assessments as useful for cost-effective monitoring between outpatient visits [28]. Further positive results and benefits from mHealth interventions have recently been summarized by Seppen et al. [31]. In line with those findings our rheumatologists, who e.g., valued the App data for permitting broader views on the course of the disease, reported some positive effect on the patient-physician interaction and on therapy adherence, and recommended its further use. Our data showed that App use for self-reporting of symptoms is feasible longitudinally, and retention rate after 3 months was still high in MiDEAR. Thus, as reported from other ePROM studies, the App use might contribute to optimized patient–doctor interactions and care [31, 32].

Similar to work from others having reported on App use in arthritis, MiDEAR additionally described needs, requirements, and liked but also disliked features from RA patients’ and physicians’ perspectives that were incorporated in the App update [33–35].

Missing data can badly affect score calculation and limit PROMs’ usefulness. The RheumaLive App minimized unintentional non-response by an error prompt. While data saving with missing values was permitted, the user was not able to see a score value if the number of missing items was too high for calculations according to the algorithms. While leaving ePROMs’ questions unanswered has been regarded as an important feature of personal choice, this opportunity was used only in the vast minority of our cases [36]. We feel this is a typical example of potential superiority of Apps that include PROMs over the traditional paper forms. In addition, Apps facilitate additional real-time, time-stamped, and long-term systematic patient-centered data collection and they might serve modern management strategies in the rigid health care systems in the next years [37–39]. A similar approach has been used with electronic diaries in clinical trials to transmit self-measured clinical parameters, and furthermore, diaries on smartphones have been reported to be convenient for users [40–42].

When applying Apps for clinical use the issues “efficacy of treatment” and “protection of patient safety” need to be raised [43]. Apps are increasingly regarded as medical devices [25, 44], and “Conformité Européenne (CE) marking” is regarded as a prerequisite before spreading an App in health care [45]. RheumaLive had been registered as a medical device to the German Institute for Medical Documentation and Information database (registration number DE/CA21/STAR Healthcare/2015/01/A/0001, document number 00132034). In order to meet the requirements for Germany’s “Digital Healthcare Act,” proof of benefit still needs to be provided [11]. Until today, no disease-specific App for RA patients meets these requirements, see digital health application (DiGA) directory (https://diga.bfarm.de/de).

When starting the proof-of-concept study, no methodology to develop health Apps for RA patients was available. Thus, RheumaLive had been developed without taking all stakeholders’ needs and requirements into account. This approach should be omitted according to the recently published EULAR points to consider for the development, evaluation, and implementation of mobile health applications [8, 26].

In general, it should be kept in mind that Apps for mobile devices are of a particular (clinical) value as these are common, carried around with the patients most of the time, and usually stick to their owners [46]. For high patients’ acceptance of ePROMs and the “conservation of instrument measurement equivalence,” the BYOD approach has been recommended [24]. However, when Apps with PROMs are commonly used, some patients will be unfamiliar with scores depicted to them requiring their education [4]. More educational content has recently also been identified for the next iteration step of an App developed by Kristjansdottir et al. [47].

For App use in routine care, it is necessary to take special needs (e.g., those of elderly, accessibility to people with higher degrees of disabilities) into account and need to be in line with further recently published recommendations [26]. Long-term data accessibility is of major concern in management of chronic diseases. The rapid progress in mobile devices and operating systems requires at least permanent updates to permit access of user data over many years. Even if interoperability of developments is said to be ensured by the manufacturers, "new" and current standards (e.g., Fast Healthcare Interoperability Resources (FHIR)) need to be taken into account. In addition, feasibility aspects are device-, operating system-, and App-specific and can only be generalized to a certain extent.

### Limitations

Our data represent data from a tertiary center; studies in larger cohorts and different clinical settings are warranted. Patients showed low and stable disease activity and good functional capacity at both investigations, data from samples with higher disease activity, and/or lower functional limitations are warranted as active arthritis may limit use of mobile devices.

The differences in age, disease duration, and education level in comparison to non-participants and to the average RA population captured in the national database indicate that younger, well-educated patients seemed to be attracted to and willing to accept the use of new technology in their own health management. The number of our patients may be too small and the deviations from the broader RA population suggest caution in generalizing the results, especially for populations with very different characteristics, e.g., social or economic marginalization such as in developing countries. Though getting less likely as we are facing more IT-savvy and well IT-equipped patients, not every RA patient might fulfill the prerequisites to use Apps (e.g., operating system to old).

## Conclusion

In MiDEAR, we demonstrated feasibility and acceptance of disease surveillance in RA by the use of established ePROMs in a smartphone App. Continuous remote monitoring of disease activity, disability, and treatment efficacy via this App apart from punctual physician visits seems feasible. In the future, the rapidly emerging use of digital health Apps promises amelioration of patients’ self-empowerment and adherence in the caring process in rheumatology. Encouraging forthcoming aspects like incorporation of passively generated data from mobile devices/sensors need further evaluation.

## Data Availability

The data are available on reasonable request.
